# Childhood rare diseases and the UN convention on the rights of the child

**DOI:** 10.1186/s13023-021-02153-0

**Published:** 2021-12-24

**Authors:** Lisa Matthews, Vaughan Chin, Marisa Taliangis, Amanda Samanek, Gareth Baynam

**Affiliations:** 1 University of Western Australia, Perth, Australia; 2 Corrs Chambers Westgarth, Perth, Australia; 3 Genetic and Rare Diseases Network, Perth, Australia; 4 Western Australian Register of Developmental Anomalies, Department of Health Western Australia, King Edward Memorial Hospital, Perth, Australia

**Keywords:** United Nations Convention on the Rights of the Child, Rare disease, Human rights

## Abstract

This letter discusses an initiative that considered the rights of a child living with a rare disease in the context of the United Nations Convention on the Rights of the Child (UNCRC). The aim was to inform laypeople on the intersection between the UNCRC and rare and undiagnosed diseases. The Project was initiated in Western Australia for a national audience, with a view that it might also provide a framework that is translatable to other jurisdictions internationally. This letter discusses some of the key themes raised by the Project and the potential for further work.

Dear Editor,

In January 2019, a small group came together in Perth, Western Australia with the purpose of better communicating the rare disease experience in childhood (Project Group). Having dealt with rare disease either as patient, medical professional, carer or advocate, members of the group had practical—although not necessarily academic—knowledge of the particular challenges. To be clear, while the group worked from its experience, the difficulties of rare disease have been catalogued elsewhere. Rare disease provides unique challenges regarding awareness in the general population, visibility in systems, difficulties of early and accurate diagnosis and development, and access to therapies [1]. With all this in mind, the group challenged itself to consider whether the United Nations Convention on the Rights of the Child (UNCRC) could be translated into the childhood rare disease experience and used as a framework for communicating this to a broader population through social media.

## Why the UNCRC?

The promise of equal dignity for all human beings has been the aim of the United Nations since its inception, almost seventy-five years ago [2]. Whilst not all countries have endorsed the UNCRC, in 1989, this document became the first legally binding instrument to cover the human rights of children worldwide [3]. However, despite the importance of the UNCRC, it arguably remains a relatively unknown document within the general population. While the UNCRC is relevant to all children, it has an especially important role in the lives of vulnerable child populations such as those living with a rare disease. However, the rarity of any singular rare disease does not reflect the substantial number of children affected by the approximately 8,000 rare diseases [4]. Although seemingly counterintuitive, rare diseases are cumulatively common, with 3.5–5.9% of the population affected [4], and 70% of them exclusively start in childhood [4, 5]. Put another way, these conditions are simultaneously rare and everywhere.

Moreover, not only do the majority of rare diseases begin in childhood, they are often lifelong, frequently associated with increased suffering and mortality, and commonly without cure [6–9]. Rare diseases are complex, chronic, often require multidisciplinary care and pose many challenges to the individuals, families, clinicians, communities, and governments involved [6–9]. The rarity of the individual diseases and failure in some jurisdictions to prioritize rare diseases as a global public health priority pose special challenges. These include acquiring scientific data and implementing and assessing clinical initiatives, resulting in gaps in diagnosis, treatment, and management guidelines [6–9]. Low and middle-income countries have these and additional challenges, resulting in healthcare systems where children with rare disease are likely to receive poorer outcomes. As rare diseases have a significant impact on global child health, recognizing the rights of these children and their families is a vital task.

### Designing the project

The UNCRC is composed of 54 articles, each declaring separate rights to be afforded to every child [3]. However, while the Project aimed to improve awareness of both rare and undiagnosed diseases by relating each of these articles to the experiences of children living with rare disease, it soon became clear that aligning the rare disease experience with each of the 54 articles would be difficult for 3 reasons:

- the 54 articles do not always sit squarely with the rare disease experience;
- that there must be no suggestion that children living with rare disease are at greater advantage (or disadvantage) than other children within the umbrella of the UNHRC; and
- that the childhood rare disease world is made up not only of affected children, but family and friends, the medical community, advocacy groups and government bodies with their own perspectives and differing levels of power. These stakeholders would also need to be considered to make the posts more representative.

However, once the key nature of the Project was distilled by the group: being the use of the UNHRC as a *framework* for considering the challenges faced by children living with rare disease then it became less important to fit squarely within each article and more important to communicate broader issues that could help with the challenges faced by these children. This flexible approach helped to convey a broader message to the intended audience being children and families living with rare and undiagnosed diseases, the medical community, and the general public.

### Project timeline and distribution

The Project was completed over 15 months within a small team comprising a medical student (LM), advocacy organization leader (AS), lawyer (MT), and clinical geneticist (GB), and consisted of 35 posts published on Facebook, culminating in the final post by Rare Disease Day 2020. Some UNCRC articles were grouped to correspond to a single rare diseases theme, so the number of posts was less than the number of UNCRC articles. Each post was bite-sized, in plain English, and released every 1–2 weeks. The posts were produced for broad dissemination and with the critical input, secretariat and support from the Genetic and Rare Disease Network (GaRDN), the local peak rare disease advocacy group in Western Australia. Other key disseminating partners included the Academy of Child and Adolescent Health (a not for profit organization dedicated to promoting the health and well-being of every newborn, child, and young person in order that they may reach their maximum potential), Rare Voices Australia (Australia’s peak organization for Rare Diseases), and the Commissioner for Children and Young People of Western Australia (a government agency committed to children and young people of Western Australia being able to live in a state where they are heard, valued, healthy, and safe).

### A selection of project themes

For the purpose of this letter, the Project Group would like to highlight four themes (and associated example posts) which aligned with particular aspects of the UNCRC: advocacy and support, healthcare, global effort, and opinion and privacy. While a full list of each post aligned to its relevant UNCRC article can be found in Table 1, a comparison of the Facebook indicators for the UNCRC posts with others on the GaRDN Facebook page during the same time indicated that posts regarding advocacy and support for children and their families living with rare disease were the most likely to raise comment and interaction from viewers.

**Table 1:** List of UNCRC series posts and corresponding UNCRC articles

UNCRC article	UNCRC series post
1	**What is this Project?** Rare diseases have a big impact on child health and wellbeing around the world. Recognizing the rights of these children, their families, and areas of unmet need is an important task. Over the next year we will look at the needs of children living with rare conditions through the scope of the United Nations Convention on the Rights of the Child (UNCRC) We hope the themes covered in each installment will create a community conversation about children and their families, and the world of rare diseases
2	**How can rare disease be a universal problem?** The United Nations Convention on the Rights of the Child (UNCRC) applies to children across the world. Rare disease can also impact any child or family regardless of their race, social status, abilities, or family history Even though each rare disease only affects a small number of people (often defined as one person in 2000), there are many rare diseases which means that throughout the world there is actually a huge number of people affected. Some estimates indicate that 6–10% of the population have a rare disease, and there is estimated to be 63,000 children living with rare diseases in WA alone The total number of people affected by rare disease creates a global and universal health issue
3	**Rare diseases are a major child health issue** Although each rare disease is rare, as a group, they are common. Up to 10% of the population may have a rare disease. Rare diseases are often associated with pain, disability, uncertainty, and early death. New information suggests that more than half of rare diseases start in childhood. This creates a large burden on children and their families very early in life To improve health and social outcomes for children and families living with rare disease, Governments must recognize that there is an unmet need. This need must be addressed with a positive policy that allows the burden of rare disease to be recognized, and the best interests of these children to be met
4	**Rights should be available to children** Children across the world live with a rare disease and often have a poor quality of life—problems in our health systems can make this worse. We need to look at how organizations can solve these problems and improve quality of life Children have rights listed in the UNCRC, and to make sure these rights are met, we need laws. To create good laws, we use frameworks to describe the problems faced by patients and families with rare disease, their doctors and other health experts and how we might solve these problems Europe is a leader in increasing awareness and change in rare disease by creating frameworks and laws. In Australia, discussions between the government and key stakeholders in the rare disease sector mean there is ongoing work to create a long-term plan to improve outcomes for people living with rare disease. This collaborative work is being led by Rare Voices Australia (RVA)
5	**Children learn to use their rights** A parent is responsible for giving children as much information about their rare disease as the child can understand and trying to reduce the impact on them. To do this, parents need to understand the information they are providing and the rights of their child It is important for medical experts to give accurate information in a way people understand, to listen to what the child needs and try to understand how the person living with a rare disease and their family feels Children with rare disease have different abilities, and it is important for governments to let parents be the expert on their children. Parents should be trusted to teach children their rights and, when needed, make decisions for them. This should mean that when the child is old enough to make decisions, they have the skills and information they need to do this
6	**Children have the right to a full life** This doesn’t relate only to the length of their life, but the quality of life they have. Children with rare disease often live with suffering and the chance of early death, so they need things like relationships, work, play, education, and enjoyment. Healthy children and children with rare disease have the same rights to these things To achieve a full life, children with a rare disease and their carers need support across many areas, including health, financial, mental health, education, etc
7	**Children have the right to a name, a nationality, a family life and a diagnosis** The right of a child to have a name and nationality can be compared to the right to have a diagnosis of their medical condition. We recognize that this is one of the challenges of living with rare disease, but an early and accurate diagnosis gives the person the best opportunity to receive the medical care they need A simple path for diagnosis is needed. Delays in diagnosis can cause mental and financial stress for both the child and their family. Until there is a diagnosis, the disease may progress and there may be an ineffective treatment that can increase this stress even more Diagnosis doesn’t mean there will be a cure, but it can provide a family with certainty and an ability to plan. Some families may never receive a diagnosis, but the opportunity to try to find one and the support for doing so needs to be given
8	**Respect the right to a name, a nationality and family ties** Governments must respect a child’s right to their identity with their name, their nationality and their family relationships. In the medical world, a ‘code’ is used to name a disease. The International Classification of Diseases (ICD) codes are often used in hospitals to name diseases In Australia, there are codes for less than 5% of rare diseases. This means that most rare diseases do not have a name. Codes are needed to get funds. If your disease has no code, this makes it difficult to get funding for medical care. Better coding for rare disease will help improve care for people living with rare disease
9	**Children have the right to a relationship with both their parents** Family unity is important for all children, especially those who have extra health needs. A family unit provides support and reassurance for a child in times of need. Rare disease can place stress on families and lead to family breakdown, support to reduce this stress is critical. If the family is separated, contact with both parents is the best outcome, if it is possible Children with rare disease are often separated from their families for health reasons such as appointments, hospital stays and procedures. This is even more significant when people live in rural and remote areas and must travel for these activities Healthcare in the home and telehealth, where appointments are done over the phone or online can reduce the impact on children
10–11	**Rare disease shouldn’t kidnap a child’s future** There is a view that receiving an incorrect diagnosis of rare disease might be like taking a child out of their own country illegally. Both things can remove the child’s future potential and opportunity. The rate of misdiagnosis of rare disease in Australia is unknown. Some studies suggest it may be up to 40–50% of rare diseases. Misdiagnosis takes away from the child their future potential by creating a cycle of incorrect treatment, management, and prognosis. Misdiagnosis also limits access to appropriate support groups and potential funding Once a diagnosis has been received, many families may not question whether the diagnosis is accurate. Even if they do question, they may not know who to approach for a second opinion. Openness by medical professionals to second opinions and pathways for parents can help reduce the potential consequences of misdiagnosis
12	**Children should be involved in decisions about their health when possible** Parents often make medical decisions for children because they are considered to have reduced decision-making capacity. Despite this, it is important to include children in decision making to a reasonable extent To make decisions about health they must provide informed consent. Informed consent is consent gained from an individual when they clearly understand the possible risks and benefits of an action or decision. Benefits and risks can be both short and long term. The information about medical procedures and treatments must be given in a way that can be easily understood and is able to be described back to a medical professional One example of this is genetic testing. Informed consent for genetic testing requires the person to have an understanding of the risks involved. Issues such as the potential for psychological distress, secondary findings, and adult-onset diseases must be discussed with parents, and, in an age-appropriate way with children
13	**Children have the right to get and share information safely** Children have the right to access and share information that does not cause them harm. This can include information about themselves, such as from genetic tests that can help identify the cause of an existing childhood condition. However, information from genetic tests that predicts the chance of an adult condition, such as Alzheimer’s Disease, could be harmful to children Adolescents may experience psychological distress whether or not they undergo testing that predicts the possibility of developing a condition as an adult. There is no ‘right’ answer about when or whether this testing should happen and it should be considered on a case by case basis following consultation between children, their parents and clinicians. Discussion about this issue is important to inform future medical guidelines and provide support and education for parents and children
14, 30	**Children have the right to Language** 2019 is the United Nations (UN) International Year of Indigenous Languages. This UN initiative seeks to raise awareness of the importance of language in daily life. Language is crucial to all people for sustaining culture, history, and tradition. Care that recognizes culture is crucial to support children and families living with rare diseases and language should be central to this. Some of other cultural factors include: Who should be in the room; who needs to be involved in decision making; does the family need a translator; and are the medical professionals respecting traditional medical approaches? A person-centered medical approach will be holistic and recognize the importance of language and culture
15	**Children have the right to rare disease support** Children living with rare disease, and their families, have the right to join groups that support them. Support groups and non-profit advocacy groups provide help to children and families living with rare disease. These groups have well-developed initiatives and networks, promote public awareness, medical care, research, and provide other helpful activities for children and families living with rare disease. Groups may be condition-specific, general support groups, or umbrella groups—that support families living with rare disease, in general. People living with rare disease in remote or isolated places are now better able to connect in online groups. These online groups can also help those with ultra-rare diseases, who may have no other contacts within their city or state. The importance of these groups and the hard work of the individuals running them cannot be overstated
16	**Children have the right to privacy** Children have the right to privacy, and in the world of rare disease, privacy can relate to children’s health data. Data sharing in rare disease is very important to help understand, diagnose, and treat individual rare disorders, and to help improve quality of life Data sharing is central to medical care and research and can mean a greater chance of diagnosis for current and future children living with rare disease. There is potential, when the number of people with any one disease is low, that there may be a chance of loss of privacy when sharing data Data sharing that maintains privacy and informed consent will help achieve the possible benefits of data sharing. Australian genetic, undiagnosed and rare disease groups support the need for data standards and systems as outlined in the Call for a National Rare Diseases Framework
17	**Children have the right to access accurate media** Information about rare diseases from the media needs to be reliable and accurate. Children living with rare conditions, and their families, need access to information that will help them live the best lives possible. It is important to raise awareness about rare diseases and responsible mass media and social media can be a way to do this. Stories need to provide facts and limit sensationalism. This will ensure that people are not more worried than they need to be and won’t make visits to the doctor or have tests that are not needed. When working with the media, rare diseases experts should encourage the media to be factual and sensible when keeping the public informed about rare diseases
18	**All children have the right to appropriate and equitable support from Government services** Rare diseases can bring extra challenges for children and their families. Health, disability, and education are all areas that families may need to be supported in. They may need to alter their homes for a wheelchair or perhaps need some changes made at school to help their child. The Government provides assistance in ways such as the National Disability Insurance Scheme. This aims to identify the support needed and helps to provide the resources required to support those needs. It is important that the Government regularly reviews their support programs, as these support services are very important to the families of children with rare diseases. Australia’s first National Strategic Action Plan for Rare Diseases is currently in development and includes a key focus on care and support, which is informed by a new report, ‘Disability and Rare Diseases: Towards Person Centred Care for Australians with Rare Diseases’
19	**Children have the right to be cared for by people who respect them** Children with rare diseases are frequently cared for, in part, by health care workers, so medical professionals must respect the religion, culture, and language of the child. Medical professionals must also make sure they are skilled enough to care for the child medically. Currently, many doctors require upskilling in the areas of rare diseases and genetics. Rare diseases may present themselves in any area of medicine, such as through a general practitioner or a pediatric emergency presentation. As such, appropriate knowledge and confidence in rare disease diagnosis and treatment is required by all doctors. Caring for the child requires both medical skills and respect for the child
20–22	**Children with rare or undiagnosed diseases are sometimes unable to be looked after by their parents** The demands of a rare disease can be too much for some families, and through no fault of their own, parents must allow others to care for their child. Family members such as grandparents may take on the role of carer, or the child may enter the foster system. The role that non-parental carers take is large, and frequently under recognized. When children with rare diseases are looked after by carers, the carers must be aware of and involved in the medical assistance required by the child. An in-depth understanding of the needs of the child must be achieved by the carer. An appropriate level of care may not be achieved in short-term homes, and as such, longer-term care should be the aim
23–24	**Children across the world are impacted by rare diseases** Some countries are better equipped to help those children than other countries. All children have the right to good quality healthcare, regardless of what state or country they are in. The United Nations also have an aim for healthy lives and well-being at all ages across the globe. When thinking about current and future goals for rare diseases it is important to think of needs larger than our own country. International teamwork is required. When one country makes a breakthrough in rare diseases, the outcomes should be shared globally. Some organizations provide global resources, which help countries all over the world with diagnosis and research. Countries such as Australia can assist by responsibly sharing data, research findings, expertise, and financial assistance. Contributing to international rare diseases policy is also important
25	**Children with rare diseases and those with undiagnosed diseases need to be reviewed regularly** Rare diseases and the research surrounding them can be fast-changing. As technology advances in genetics, and research is shared across the world, rare diseases are becoming better recognized and treated. These fast changes provide hope for improved testing and treatment in the future. As scientific literature surrounding conditions changes, people with these rare diseases need to be reviewed Regular review is also significant for those children with undiagnosed diseases. Although a diagnosis may not have been reached when the child was first assessed, it does not mean that a diagnosis will not be provided in the future. By regularly reviewing children with rare and undiagnosed diseases, it may provide new diagnoses, treatments, or other management options
26	**Family stress due to demands of rare disease and lack of financial help** Living with rare disease can contribute to significant increased stress on families, and financial burden can further this stress. Financial hardship in the families of children with rare diseases may worsen an already difficult situation. The additional costs for these families can include specialized equipment, loss of income for caregivers, and specialized medical or allied health services. Children with undiagnosed diseases may also have additional costs due to extra investigations and appointments. Costs may vary depending on the condition and the experience of the family. Costs may be hidden and present themselves slowly over childhood. Costs may also differ depending on whether the individual living with rare disease uses public or private services. Although some financial aid may be given through programs such as the NDIS, families may still need of extra financial assistance
27	**Family stress due to excess demands** Children with rare disease have the right to have both their physical and mental needs looked after. It is important to consider the mental health of children and their families living with rare disease because they can experience high levels of stress. We need to make sure that accessing services does not make this stress worse. It can take a long time to diagnose a rare disease, and even after diagnosis can be hard to find information or a specialist who knows about the disease. Health professionals need to be aware of how important mental health support is for rare disease families. Educating health professionals about the resources and support groups available to families living with rare disease is also essential to reduce the mental stress of living with rare disease
28	**Access to an education is a basic right for every child** We need to make sure children with complex health issues are given the support they need to thrive and reach their potential in education. This includes social, emotional, and physical needs as well as their academic needs Teachers need support in how to cater to children with rare disease and how they can help families to access support services When a child isn’t able to access mainstream education, it is important that they continue not only to access the curriculum, but also to stay connected with their classmates and teachers, and to continue achieving their goals, and being challenged and stimulated away from the traditional classroom
29, 31	**Learning through play** We often hear about ‘Learning through play’ in education, this is important for all children and especially for children living with rare disease. Children should feel safe, secure, and confident to share their thoughts and feelings, and to learn through discovery. Play gives children the chance to connect with other children and adults, which is especially important for children living with rare disease, who may spend a lot of time outside the classroom. Play-based learning helps children to develop self-esteem, problem-solving skills and coping skills, and gives some children the chance to work through any trauma they have experienced while living with their condition. Play gives children the chance to grow emotionally as well as academically
32, 34, 37	**Children with rare diseases must be protected from harm and exploitation** Research is very important to help find ways to treat and manage rare disease, but it is important that children with rare disease and their families are not taken advantage of. When families are desperate to help their child, they are more likely to take risks, such as experimenting with overseas research or treatment. It is important when a child or their family take part in research that there isn’t a safer or more reliable alternative, that the treatment can help the child, and that they are either paid for participating or have their costs covered. Being involved in research should not have a negative impact on the health of the child or the finances of the family. It is important that health professionals are informed about the risks and benefits of being involved in medical trials or treatments and are available and willing to explain these to the children and families at risk
33	**Rare disease and drug treatment** Children with rare disease often need drug treatment as part of their care. Drugs to treat rare disease can be rare ‘orphan drugs’ and many are still in their trial phase when they are used to try and treat people with rare diseases. Sometimes this means that not all of the harms and benefits are known while the drugs are being used in the trial phase. As many rare diseases are progressive, it is especially important that children have timely access to these drugs as they can lead to improvements for people living with rare disease. It is critical that the use of orphan drugs is governed by strong laws and oversight to make sure that the benefits can be harnessed, and any potential harm is limited
35	**Equal access to medical services and research** Children with a rare disease can have complex medical needs that can be difficult to manage within the public health system in Australia. Wait times to see private doctors can be long and this can result in families feeling that they have no choice but to pay for services in the public sector or be involved in research trials to get the best treatment in a timely manner. It is critical to ensure that all Australians have equal access to medical services and research that will best support their health and that communication supports people making the choices that are best for their child with a rare disease
36	**Transitions in care must be supported** Many children with rare disease can ‘fall through the cracks’ of systems as they move from child to adult services. This period is challenging for most children but those with rare diseases have more to contend with. Where possible, children should be encouraged to engage more in their own care, to understand their condition and how to manage it—this may require additional support. The rare disease community has clearly stated that there needs to be better support for the transition from child to adult services. Hospitals across the country are starting to do more to create these services, including the transition clinic for complex and rare disorders at Perth Children’s Hospital that opened in 2019. This clinic provides a path to support the health transition. More is needed in the disability, education, employment, and other sectors to support the development of the whole person as they live with rare disease
38	**Rare disease research may help all** The battlefield of rare diseases is a short paper that looks at how rare disease research can improve things for the whole population. The paper reviews the work of Goldstein and Brown, who did research on familial hypercholesterolemia, a rare disease that can increase the risk of heart disease. This research later resulted in statins being developed that are now used regularly to help prevent heart and artery disease, and the researchers won a Nobel prize for their work. This is just one example of how research in rare disease can lead to greater scientific understanding and improve life for the whole population. Funding for rare disease research can have outcomes that reach far beyond the rare disease community
39–40	**Right to rescue: extra help for children** Children with rare diseases may need extra help to develop their self-esteem and self-respect. These children might be seen as different because of their rare disease. They may not have a diagnosis or it may take a long time to get a diagnosis. They can have greater physical needs. They may have trouble with social settings. All these things can impact their mental health. They need more support from services and peer support groups. Recognizing their experience is an important first step and creating pathways that focus on the quality of life, self-respect, social integration, and access to more support need to be identified, developed, and promoted
41	**National Action for Rare Disease** Care and services for children living with rare conditions vary from country to country. Some places have more support, better care, and laws that are models for improving the lives of children living with a rare disease. Some countries have National Rare Disease Plans that address equitable access to care and treatment, programs for diagnosis, care coordination, research, and engagement and support Australia has recognized this need and today, the Federal Health Minister, the Hon Greg Hunt MP, launched the National Strategic Action Plan for Rare Diseases. Rare Voices Australia has led the collaborative development of the Action Plan that has been developed by the rare disease sector, for the rare disease sector. The Action Plan works towards the best possible health and wellbeing outcomes for Australians living with a rare disease
42–54	**Rare Aware** The final part of the United Nations Convention on the Rights of the Child focuses on the fact that everyone should know about this Convention so that people can understand their rights and advocate for themselves and their child to ensure they have the best possible quality of life. The same applies to rare disease—everyone needs to know about rare diseases. This makes it easier for children living with a rare disease and their families and carers to get the care, support and understanding they need. Our hope for the future is that more people understand rare diseases, including medical professionals, policymakers, and lawmakers, so that new technology, research, and global collaborations can give children living with a rare disease the best chance of a long and full life

### Right to advocacy and support

Multiple articles within the UNCRC point to the right to advocacy and support for both the parents and the child. For example, Article 3 states that all organizations concerned with children should work towards what is best for the child and Article 26 refers to governments providing monetary support to families in need [3]. Similarly, children with rare disease may need additional advocacy and support in areas such as mental health, physical health, and financial health. The following post addressed financial impact:

Living with rare disease can contribute to significant increased stress on families, and financial burden can further this stress. Financial hardship in the families of children with rare disease may worsen an already difficult situation. The additional costs for these families can include specialized equipment, loss of income for caregivers, and specialized medical or allied health services. Children with undiagnosed diseases may also have additional costs due to extra investigations and appointments. Costs may vary depending on the condition and the experience of the family. Costs may be hidden and present themselves slowly over childhood. Costs may also differ depending on whether the individual living with rare disease is using public or private services. Although some financial aid may be given through programs such as the NDIS*, families may still be in need of extra financial assistance.

*National Disability Insurance Scheme

## Right to healthcare

Healthcare can be non-existent or inaccessible for children with rare disease, whether for financial, geographic, cultural or other reasons. Healthcare challenges can be further complicated by the absence of a diagnosis. Accordingly, the international recommendations to address the specific needs of undiagnosed people have been developed [10]. The UNCRC supports this right to healthcare in Article 6, determining that governments should ensure every child has a full life to survive and develop healthily where possible, and in Article 23, stating that children who have any kind of disability should receive special care and support so that they can live a full and independent life [3].

A key barrier to rare disease healthcare is funding. This applies not only to government funded diagnostics and treatment, but also to funding for research to create these diagnoses and treatments [11]. We cited a topical piece, The Battlefield of Rare Diseases [12], to address this concern and demonstrate how advances in rare disease may contribute to better healthcare for all disorders:

The battlefield of rare diseases is a short paper that looks at how rare disease research can improve things for the whole population. The paper reviews the work of Goldstein and Brown who did research on familial hypercholesterolemia, a rare disease that can increase the risk of heart disease. This research later resulted in statins being developed that are now used regularly to help prevent heart and artery disease and the researchers won a Nobel prize for their work. This is just one example of how research in rare disease can lead to greater scientific understanding and improve life for the whole population. Funding for rare disease research can have outcomes that reach far beyond the rare disease community.

## Right to global effort

The Project Group believes that one of the most important issues is the need for a global effort in the fight against rare diseases. Strength in numbers, and solidarity, are often required in medicine and human rights, and to achieve scale and unity requires global cooperation. Articles 24 and 28 both address this concept, recognizing that better-resourced countries should support the needs of less-resourced countries to achieve the overall goals of upholding children’s rights [3]. Similarly, international collaboration is required in response to rare diseases, and for optimal impact, a collaboration involving epidemiological and genetic data, and implementation and assessment of clinical initiatives [11, 13].

Children across the world are impacted by rare diseases. Some countries are better equipped to help those children than other countries. All children have the right to good quality healthcare, regardless of what state or country they are in. The United Nations also have an aim for healthy lives and well-being at all ages across the globe. When thinking about current and future goals for rare diseases it is important to think of needs larger than our own country. International teamwork is required. When one country makes a breakthrough in rare diseases, the outcomes should be shared globally. Some organizations provide global resources, which help countries all over the world with diagnosis and research. Countries such as Australia can assist by responsibly sharing data, research findings, expertise, and financial assistance. Contributing to international rare diseases policy is also important.

## Right to an opinion and right to privacy

The right to an opinion, together with a right to privacy, are particularly relevant in rare disease in the instances of opting out of genetic testing or opting into research trials, and the data resulting from these initiatives [14, 15]. Article 12 addresses children’s right to voice what they think should happen when adults are making decisions that affect them, and Article 13 asserts that children have the right to obtain and share appropriate information [3]. The following post demonstrates the added complexity of the right to privacy, mentioned in Article 16 [3], in the context of the specialized data needed with rare diseases.

Children have the right to privacy, and in the world of rare disease, privacy can relate to children’s health data. Data sharing in rare disease is very important to help understand, diagnose, and treat individual rare disorders, and to help improve quality of life.

Data sharing is central to medical care and research and can mean a greater chance of diagnosis for current and future children living with rare disease. There is potential, when the number of people with any one disease is low, that there may be a chance of loss of privacy when sharing data. Data sharing that maintains privacy and informed consent will help achieve the possible benefits of data sharing. Australian genetic, undiagnosed and rare disease groups support the need for data standards and systems as outlined in the Call for a National Rare Disease Framework.

## Conclusion

The UN High Commissioner for Human Rights has identified persons living with rare diseases as a particular area of focus, being a high needs population that are usually excluded [16]. Against this, the Project Group was able to successfully apply the UNCRC as a framework to consider the rights of children living with a rare disease and communicate this to the larger population. In this way, the UNCRC provided a scaffold to advance the message that rare diseases of childhood impact not only health, but also, fundamental human rights. Although this Project focused on the Australian context, it is believed that this approach is globally generalizable, with tailoring to individual countries or regions. Whether this methodology can underpin policy design or specifically articulated rights for rare disease patients is outside the scope of this letter but the alignment of the rare disease experience with human rights does commend itself to further research.

## Data Availability

Not applicable.

## Abbreviations

UNCRC: United Nations Convention of the Rights of the Child
GaRDN: Genetic and Rare Disease Network
NDIS: National Disability Insurance Scheme
